# Retraction Note: RNA editing of BFP, a point mutant of GFP, using artificial APOBEC1 deaminase to restore the genetic code

**DOI:** 10.1038/s41598-025-89490-3

**Published:** 2025-02-11

**Authors:** Sonali Bhakta, Matomo Sakari, Toshifumi Tsukahara

**Affiliations:** Area of Bioscience and Biotechnology, School of Materials Science, Japan Advanced Institute of Science and Technology, M1-4F, 1-1 Asahidai, Nomi City, Ishikawa 923-1292 Japan

Retraction of: *Scientific Reports* 10.1038/s41598-020-74374-5, published online 14 October 2020

The Editors have retracted this Article. Concerns were originally raised about Fig. 3A, which was subsequently corrected^1^. Following the correction, additional concerns were identified:

- In the corrected Fig. 3A, the electrophenogram trace contains discontinuous lines that end abruptly;
- The trace shown in corrected Fig. 3A does not appear to match the original plasmid sequence;
- The corrected version of the Article includes a new Supplementary Fig. 6 which appears to acknowledge the off-target editing event that was initially shown in the original Fig. 3A as the targeted editing event. However, it has not been acknowledged that the targeted and untargeted editing sites are only five nucleotides apart, and the two events are shown on separate traces rather than on a single trace. Furthermore, the corrected Article still contains the statement that no off-targeting event had occurred within 21 bp upstream or downstream of the target.
- The original Fig. 3B has been removed from the corrected Fig. 3 that now only shows data from the forward primer. The article, however, still mentions findings with the reverse primer.

The Authors did not provide an explanation for these concerns nor the original data. The Editors, therefore, have lost confidence in the results reported in the Article. All authors agree to this retraction.
